# Copper decorated Ti_3_C_2_ nanosystem with NIR-II-induced GSH-depletion and reactive oxygen species generation for efficient nanodynamic therapy

**DOI:** 10.1515/nanoph-2022-0599

**Published:** 2022-11-04

**Authors:** Yuanyuan Zhang, Shuang Li, Xueyang Fang, Beiping Miao, Yujie Wang, Jiantao Liu, Guohui Nie, Bin Zhang

**Affiliations:** Shenzhen Key Laboratory of Nanozymes and Translational Cancer Research, Department of Otolaryngology, Shenzhen Institute of Translational Medicine, The First Affiliated Hospital of Shenzhen University, Shenzhen Second People’s Hospital, Shenzhen 518035, China; State Key Laboratory of Chemical Oncogenomics, Guangdong Provincial Key Laboratory of Chemical Genomics, Peking University Shenzhen Graduate School, Shenzhen 518055, China; Department of Orthopedics, The First Hospital of Xi’an Jiaotong University, Xi’an 710061, China

**Keywords:** GSH-depletion, nanodynamic therapy, NIR-II-induced phototherapy, reactive oxygen species

## Abstract

Nanodynamic therapy (NDT) based on reactive oxygen species (ROS) production has been envisioned as an effective cancer treatment. However, the efficacy is limited by the hypoxia, insufficient hydrogen peroxide conversion, and high glutathione (GSH) levels in the tumor microenvironment (TME). To solve these issues, we proposed and designed a biocompatible, oxygen resistant Cu-modified Ti_3_C_2_ nanocomposite (Ti_3_C_2_-Cu-PEG), which can efficiently deplete the endogenous GSH in tumor cells, smartly respond to NIR-II light irradiation with in-depth tissue penetration to achieve photothermally enhanced tumor photodynamic therapy (PDT) and catalytic therapy. Specifically, Ti_3_C_2_-Cu-PEG reacted with oxygen to produce singlet oxygen (^1^O_2_) under NIR-II irradiation, and catalyzed the highly expressed H_2_O_2_ in the tumor microenvironment to generate ·OH. In addition, Ti_3_C_2_-Cu-PEG significantly decreased intracellular GSH, reduced the chances of reaction between ROS and GSH, and thus promoting ROS effect. Moreover, the intrinsically high photothermal conversion efficiency of Ti_3_C_2_-Cu-PEG further promotes the NDT process. *In vitro* and *in vivo* experiments, the Ti_3_C_2_-Cu-PEG nanosystem showed excellent antitumor effect in 4T1 tumor-bearing mice by amplifying oxidative stress under NIR-II stimulation. This work highlights an easily synergistic nanosystem with remodeling TME and combined photothermal therapy to enhance the therapeutic effect of NDT in tumor therapy.

## 1 Introduction

Reactive oxygen species (ROS) plays a key role in maintaining various signaling pathways and normal physiological processes [1]. Compared with normal cells, tumor cells have high ROS levels in the tumor microenvironment due to aberrant proliferation and metabolism [2], which is also accompanied by upregulation of antioxidant defense systems glutathione (GSH) and further contributes to the maintenance of “healthy” level of ROS for self-preservation [3]. The altered redox stability not only contributes to tumor development and progression, but also increases the susceptibility of tumor cells to oxidative damage [4]. Nanodynamic therapy (NDT), including photodynamic therapy (PDT), thermodynamic therapy (TDT), sonodynamic therapy (SDT), electrodynamic therapy (EDT), radiodynamic therapy (RDT), and chemodynamic therapy (CDT), based on highly toxic ROS (·OH, ^1^O_2_, etc.) generation to break the redox balance in tumor cells has been envisioned as a distinct modality for efficient cancer treatment [5–9]. The key mechanism of NDT lies in production of versatile reactive species assisted by nanomaterials, such as toxic radicals, for inducing apoptosis or necrosis by destroying cellular components including proteins, lipids, or nucleic acids, thus avoiding drug resistance which is common in conventional chemotherapy [10, 11]. In addition, nanodynamic therapy can specifically exploit the tumor microenvironment (low acid, high H_2_O_2_, or GSH) to produce highly toxic ROS, leaving the normal cells or tissues undamaged, which might be regarded as a minimally invasive and intelligent therapy. As a result, their therapeutic efficacy is relatively high, in accompany with far fewer side effects. Therefore, the elevation of ROS in tumor microenvironment (TME) by scavenging ROS or increasing ROS generation presents a new era for tumor therapy [12, 13].

Currently, the most reported nanodynamic therapies are PDT [14–19] and CDT [20–22], with the former applied in clinic therapy of superficial tumors for decades [7, 23]. The principle of PDT in clinical applications is to use light of specific wavelength to irradiate photosensitive drugs gathered at tumor site, so that it can transfer energy to the surrounding oxygen, thus generating highly active ^1^O_2_ and killing tumor cells [24]. CDT essentially rely on Fenton or Fenton-like reaction to catalyze less harmful hydrogen peroxide (H_2_O_2_) in acidic condition into ·OH, the most toxic ROS, to induce cancer cell damage and apoptosis [25]. The three essential factors of CDT process are an acid environment, H_2_O_2,_ and transitional metals. Thereby, taking advantage of the two characteristics of acidity and endogenous H_2_O_2_ overexpression of tumor microenvironment (TME), CDT has been widely explored as a more specific therapy against cancer with no dependence on either oxygen or external energy input, and concurrently without considering the restrictions of the penetration depth of laser through tissues [26, 27]. Photodynamic therapy and chemodynamic therapy are two reactive oxygen species (ROS)-induced cancer cure strategies, aiming at killing cancer cells with excessive ROS, such as singlet oxygen (^1^O_2_) and hydroxyl radical (·OH), but are still limited by their inherent limitations [28]. Conventional PDT is limited by laser penetration depth (visible or NIR-I light is most used) and tumor hypoxic environment, while CDT is limited by acidity and H_2_O_2_ concentration. Compared with visible or NIR-I light, the penetration depth of NIR-II light (1000–1300 nm) is significantly improved [29, 30]. The development of photosensitizer based on NIR is very important for improving photodynamic therapy. Photothermal therapy induced by light can promote blood circulation in the tumor site and alleviate tumor hypoxia to a certain extent [31, 32]. Thermodynamically, increased temperature is beneficial for catalytic process in principle, thus improving the production efficiency of ·OH in Fenton reaction to strengthen the anti-tumor effect. The preparation of nanosystems integrating PDT, CDT, and PTT is of great significance for smartly harnessing the complex microenvironment of tumors and effectively inhibiting tumors [33, 34].

In recent years, MXene nanosheet is an ideal support candidate and has been widely used in tumor phototherapy due to its large specific surface area with uniform composition and easy functionalization, broad light absorption, high photothermal conversion efficiency and good biocompatibility [35–40]. MXene nanosheets, especially Ti_3_C_2_, are usually employed for loading drugs or metal nanoparticles to achieve combined therapy, but suffer from poor stability against O_2_ and water [41, 42]. Among the reported CDT reagents, the Cu-mediated Fenton-like reaction is more likely to occur, catalyzing H_2_O_2_ to highly toxic ·OH, independent of weakly acidic pH, and is 160 times faster than Fe due to the lower redox potential of Cu^+^/Cu^2+^ [43–45]. Moreover, Cu^2+^ can oxidize GSH into GSSG, leading to depletion of GSH and breakage of redox balance a step further [46]. Cancer cells always have high levels of glutathione (GSH) to maintain their antioxidant system, and GSH will consume reactive oxygen species (ROS) produced by PDT, which will significantly reduce the efficacy of PDT [47–49]. In terms of these issues, combination of positively charged Cu species with MXene may provide a promising nanoplatform for admirable tumor therapy.

**Scheme 1:**
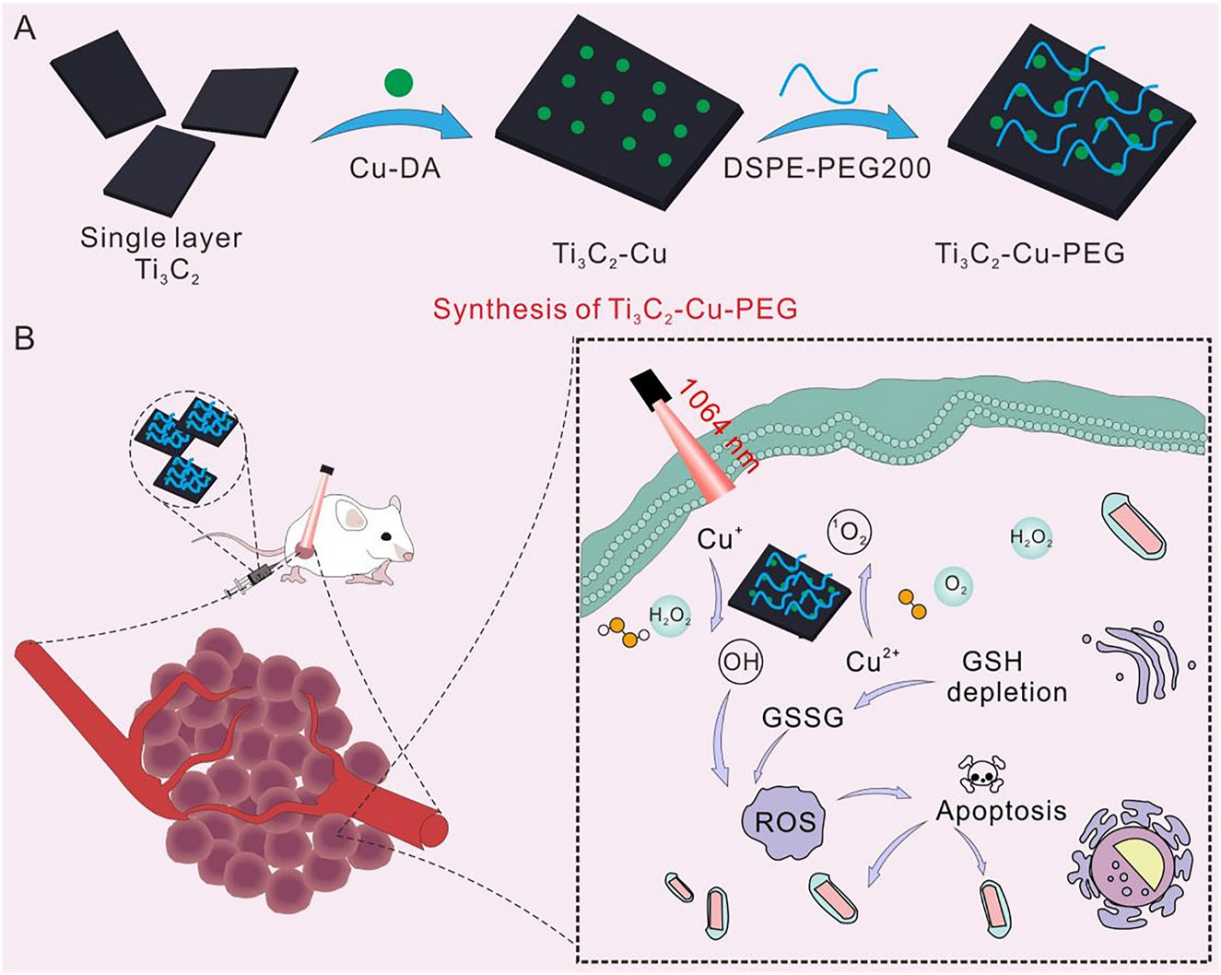
Schematic illustration of the synthesis process of Ti_3_C_2_-Cu-PEG (A) and the nanoplatform depleted the GSH in TME and achieved NIR-II induced PTT together promoted PDT and CDT for efficient synergistic cancer therapy.

Herein, we successfully developed a Cu-decorated Ti_3_C_2_ nanocomposite (Ti_3_C_2_-Cu-PEG) where Cu species were evenly dispersed on the Ti_3_C_2_ surface. This nanoplatform successfully depleted the GSH in TME and achieved NIR-II light-induced PTT together with thermal-promoted PDT and CDT for efficient synergistic cancer therapy (Scheme 1). On the one hand, in-depth PDT could be realized in the deeper layers of the tumor under NIR-II irradiation, and hypoxia was alleviated dramatically due to the PTT, further enhancing the efficacy of PDT. On the other hand, CDT induced by Cu was enhanced under the promotion of heat and special pH in TME, which improved the therapeutic effect and synergistically killed cancer cells. In addition, because Cu^2+^ depletes GSH, our material broke the redox balance of tumor microenvironment, generates more ROS, and promotes the efficacy of PDT and CDT. Finally, we systematically evaluated and demonstrated the biosafety and therapeutic properties of the materials for tumor ablation *in vitro* and *in vivo*.

## 2 Experimental section

### 2.1 Synthesis of Ti_3_C_2_-Cu-PEG nanocomposites

Firstly, copper acetate and dopamine hydrochloride were combined to form a complex with a molar ratio of 1:3 (Cu/DA), so that copper ions could be better adsorbed to Ti_3_C_2_ nanosheets. Next, the Cu-DA complex was loaded onto Ti_3_C_2_ nanosheets by electrostatic adsorption. First, the purchased Ti_3_C_2_ nanosheet suspension (10 mg) was dispersed in Tris buffer (5 mL) and sonicated in a water bath for 10 min to form a homogeneous solution. Under strong magnetic stirring, the prepared Cu-DA solution was slowly dropped into Ti_3_C_2_ solution and stirred for 2 h. Then, DSPE-PEG2000 (10 mg) DMSO solution was added to the above solution, stirred for 2 h, and dialyzed for 12 h to remove impurities. Finally, Ti_3_C_2_-Cu-PEG nanocomposites were concentrated in ultrafiltration tubes and dispersed in water, and then refrigerated for later use.

### 2.2 *In vitro* photothermal performance of Ti_3_C_2_-Cu-PEG

To evaluate the photothermal performance, a 1064 nm laser (1 W/cm^2^) was used to irradiate the Ti_3_C_2_-Cu-PEG aqueous solution for 300 s at room temperature; the temperature change and thermal image at different time points were photographed and recorded with an IR thermal camera. As a comparison, PBS and Ti_3_C_2_-PEG was irradiated under the same conditions. Ti_3_C_2_-Cu-PEG aqueous dispersion with various concentrations (0–100 μg/mL) was irradiated with 1 W/cm^2^ by the 1064 nm laser for 5 min. Meanwhile, the temperature changes of Ti_3_C_2_-Cu-PEG (100 μg/mL) were also measured under the irradiation of 1064 nm laser with various powder densities. The Ti_3_C_2_-Cu-PEG aqueous solution was irradiated at 1 W/cm^2^ to calculate the photothermal conversion efficiency, and the change in temperature every 30 s was recorded to test the photothermal stability of the material (100 μg/mL) for four cycles.

### 2.3 *In vitro* ROS production

#### 2.3.1 Evaluation of ·OH generation by TMB

To detect the production of ·OH, 3,3′,5,5′-tetramethylbenzidine dihydrochloride (TMB) was used as a probe by the color reaction, which can be oxidized by ·OH to blue oxTMB and increase the absorption intensity of TMB at 650 nm. The spectrum was measured using a UV–vis spectrophotometer. Ti_3_C_2_-Cu-PEG nanocomposites were mixed with TMB and H_2_O_2_ in PBS solution. The change of the solution absorbance was recorded by the UV−vis spectrophotometer. And under the same conditions, the sample was dissolved in PBS at different H_2_O_2_ concentrations and the influence of 1064 nm laser was compared.

#### 2.3.2 Evaluation of ·OH generation by MB

A total of 100 μL of Ti_3_C_2_-Cu-PEG nanocomposites was added in MB aqueous solution containing H_2_O_2_. After incubation for different time intervals, the Ti_3_C_2_-Cu-PEG nanocomposites were removed from the MB aqueous solution by centrifugation. Then the UV−vis spectrophotometer was adopted to measure the absorbance of the supernatant at 664 nm.

#### 2.3.3 Singlet oxygen (^1^O_2_) generation

As the singlet oxygen (^1^O_2_) indicator, 1,3-Diphenylisobenzofuran (DPBF) could react with ^1^O_2_ under irradiation with a 1064 nm NIR laser (1 W/cm^2^) resulting the decrease for DPBF absorption at about 410 nm. Typically, DPBF were mixed with Ti_3_C_2_-PEG or Ti_3_C_2_-Cu-PEG nanocomposites solution (100 μg/mL) was irradiated under 1064 nm laser with power density of 1.0 W/cm^2^ at different time point over 30 min, and then the spectra were recorded.

### 2.4 *In vitro* GSH depletion

The depletion of GSH was monitored by UV−vis spectroscopy. 5,5′-Dithiobis-(2-nitrobenzoic acid) (DTNB) was used to detect GSH in solution. DTNB can react with the -SH group on GSH to form 2-nitro-5-thiobenzoate anion (TNB^−2^), which appears bright yellow at 412 nm. Ti_3_C_2_-Cu-PEG aqueous solution were mixed with DTNB and GSH solutions under 1064 nm laser (1 W/cm^2^, 5 min) and then the UV–vis spectroscopy was applied to detect the absorbance of the above suspension.

### 2.5 Intracellular GSH content

4T1 tumor cells were seeded into a 6-well plate for allowing attachment. Then, these 4T1 tumor cells were co-incubated with different concentration of Ti_3_C_2_-PEG or Ti_3_C_2_-Cu-PEG (100 μg/mL) for 20 h and treated with 1064 nm laser (1 W/cm^2^, 5 min). After 4 h, these cells were collected and washed with PBS three times and then incubated with 0.5 mL Triton X-100 (1%) for 2 h at 4 °C, the lysate was centrifuged at 10,000 rpm for 10 min. The supernatants were collected for GSH analysis via GSH and GSSG assay kit.

### 2.6 Intracellular ROS generation detection

2,7-dichlorodihydro-fluorescein diacetate (DCFH-DA) was used as the probe to detect intracellular ROS. 4T1 cells were seeded into culture dishes for 20 h (37 °C, 5% CO_2_). Then, Ti_3_C_2_-PEG or Ti_3_C_2_-Cu-PEG nanocomposites medium solution (100 μg/mL) was added and incubated for 4 h. Then, the cells were exposed to a 1064 nm laser (1 W/cm^2^) for 5 min. After that, the cells were washed with PBS. DCFH-DA solution was added to the cells for 10 min. The strength of green fluorescence was obtained by Echo Laboratories Revolve FL by an excitation of 488 nm.

### 2.7 Mitochondrial integrity assay

4T1 cells were cultured in six-well plates for adherent growth and incubated for 24 h. Subsequently, the cells were incubated with Ti_3_C_2_-PEG or Ti_3_C_2_-Cu-PEG nanocomposites. After 4 h of incubation, the cells exposed to a 1064 nm laser (1 W/cm^2^) for 5 min. The medium was removed and replaced with JC-1 staining solution according to the manufacturer’s protocol. The cells were washed 3 times with PBS and imaged by Echo Laboratories Revolve FL.

### 2.8 Cytotoxicity assay

The cytotoxicity analyses of different samples were performed via cell-counting kit 8 (CCK-8) assay. HUVEC, NIH3T3, and 4T1 cells were seeded in 96-well plates. After 24 h cultivation at 37 °C, the medium was replaced by the medium containing Ti_3_C_2_-PEG or Ti_3_C_2_-Cu-PEG nanocomposites at a serial of different concentrations (12.5, 25, 50, and 100 μg/mL). After 4 h incubation, the wells of 4T1 cells in different groups were irradiated by a 1064 nm laser for 5 min. All of the groups were incubated for another 20 h and then all the wells were incubated with CCK-8 solution for 4 h. After that, the plate reader was used to record the absorbance at 450 nm.

### 2.9 Live/dead cell staining assay

The calcein-AM and propidium iodide were used to evaluate the live cells and dead cells, respectively. 4T1 cells were seeded in a six-well plate with a density of 1.0 × 10^5^ cells per well and incubated with Ti_3_C_2_-PEG or Ti_3_C_2_-Cu-PEG nanocomposites (100 μg/mL). As for laser irradiation groups, all of the treatment was similar to that above and irradiated by a 1064 nm laser for 5 min. After all the treatment, 4T1 cells were stained by calcein-AM and PI and then imaged by Echo Laboratories Revolve FL.

### 2.10 Apoptosis detection assay

The apoptosis-mediated cell death was quantitatively analyzed by a flow cytometer. The cultured cells were seeded in a six-well plate evenly and cultivated for 24 h. Subsequently, the 4T1 cells were incubated with Ti_3_C_2_-PEG or Ti_3_C_2_-Cu-PEG nanocomposites at the same concentration (100 μg/mL) for 4 h. The laser irradiation groups were illuminated by a 1064 nm laser (1 W/cm^2^, 5 min). Then, all of the treated cells were trypsinized, washed, and analyzed with a flow cytometer.

### 2.11 Animal model

Female Balb/c mice (16–18 g, 5–6 weeks old) were purchased from the Laboratory Animal Center of Vital River Laboratory Animal Technology Co., Ltd. (Guangzhou, China). 2 × 10^6^ 4T1 cells were subcutaneously injected into the right back of Balb/c mice. When the tumor became distinct and the tumor volume reached about 100 mm^3^, the mice were randomly assigned into either control or test groups.

### 2.12 *In vivo* infrared thermography and therapy

The 4T1 tumor-bearing mice were randomly divided five groups (*n* = 5). Then, the different groups were administered appropriate treatments as follows: (1) PBS, (2) PBS + 1064 nm laser, (3) Ti_3_C_2_-Cu-PEG, (4) Ti_3_C_2_-PEG + 1064 nm laser, and (5) Ti3C2-Cu-PEG + 1064 nm laser. The treatment groups were administrated by intratumor injection of PBS, Ti_3_C_2_-PEG, and Ti_3_C_2_-Cu-PEG, respectively. The injection dose was controlled at 15 mg/kg. The tumor site was irradiated upon a 1064 nm laser for 5 min after 4 h of injection. The real-time temperature changes at the tumor region were recorded by an infrared thermography. Drug administration and irradiation treatment were performed only once. The change of body weight and the volume of the tumors (tumor volume = length × width^2^/2) of tumor-bearing mice were measured using a vernier caliper and an electronic balance every 2 days. After 14 day treatment, the mice were sacrificed and their tumor tissues were harvested and weighed. The tumors for all of the groups were photographed for comparison and tissues of major organs were subjected to H&E staining. To evaluate the histological damage to the tumors induced by drug and laser irradiation, one mouse from each group was sacrificed, and tumor tissues were subjected to H&E staining, TUNEL and Ki67 antibody staining at 24 h post laser irradiation.

### 2.13 Statistical analysis

The data are presented as the mean ± standard deviation (SD), and the statistical significance between two groups of data in this work was analyzed on the basis of two-tailed Student’s *t*-test (*p < 0.05; **p < 0.01; ***p < 0.001).

## 3 Results and discussion

### 3.1 Synthesis and characterization of nanocomposites

**Figure 1:**
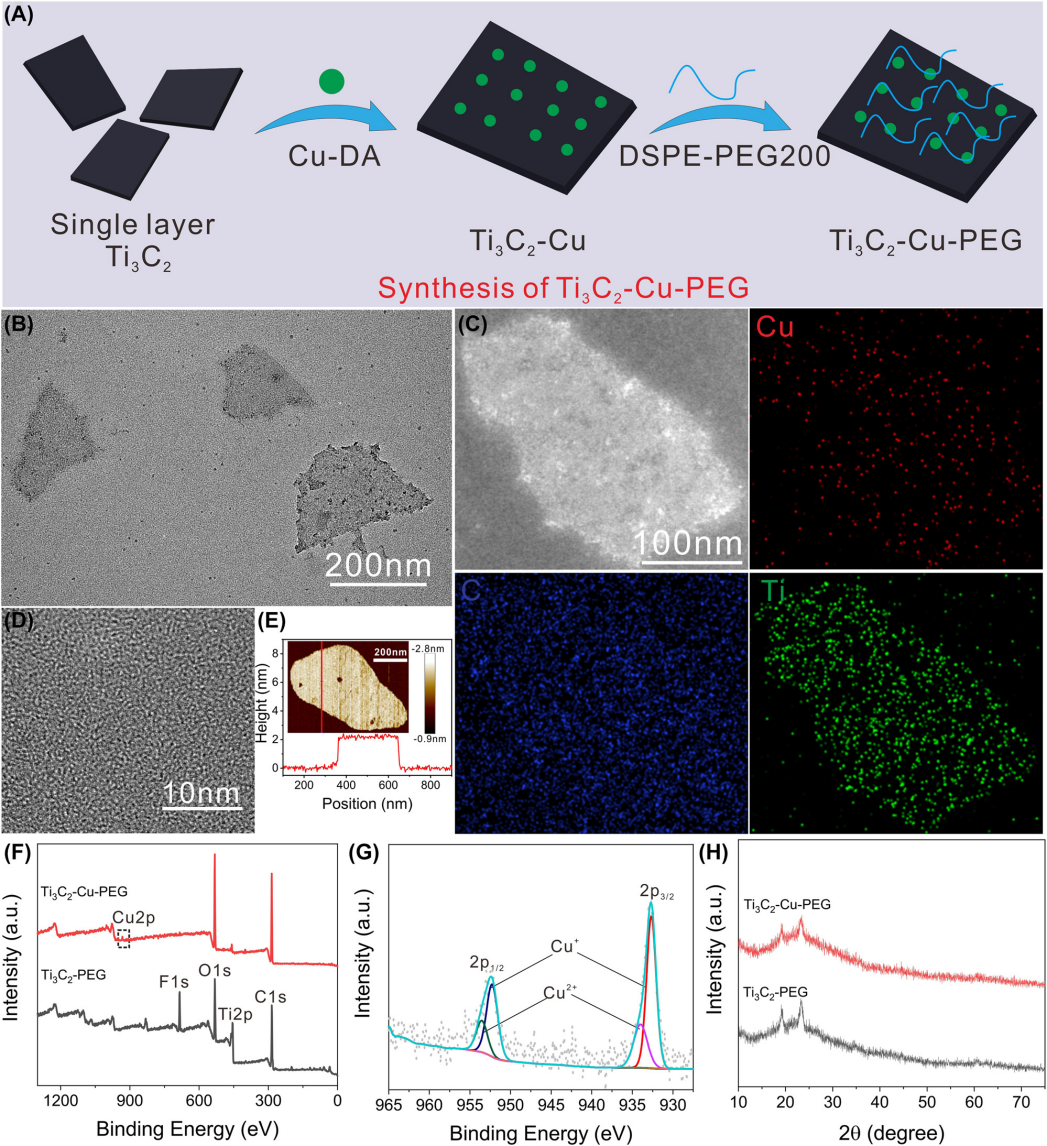
Preparation and characterization of the Ti_3_C_2_-Cu-PEG. (A) Schematic illustration of the synthesis of Ti_3_C_2_-Cu-PEG nanocomposites. (B) TEM image of single layer Ti_3_C_2_-Cu-PEG. (C) Elemental mappings images, and HAADF-STEM images (D) of Ti3C2-Cu-PEG. (E) was measured by atomic force microscopy (AFM). (F) Full X-ray photoelectron spectroscopy (XPS) analysis spectrum of Ti_3_C_2_-Cu-PEG and high-resolution profile of Cu2p (G). (H) XRD patterns of Ti_3_C_2_-PEG and Ti_3_C_2_-Cu-PEG.

Ti_3_C_2_ is one of the most widely studied members of the MXene family, which is often explored for photo-mediated tumor therapy due to its broad light absorption and excellent photothermal conversion efficiency upon irradiation of NIR light [50]. However, the ROS generation performance over Ti_3_C_2_ via PDT process is very low, so Ti_3_C_2_ nanosheet was mainly used in photothermal assisted tumor therapy. In this study, Cu cations, which have demonstrated exciting potential for catalytic conversion of H_2_O_2_, were grafted onto the surface of Ti_3_C_2_ via electrovalent bond to perform photothermal enhanced ROS-mediated tumor therapy. Adopting an electrostatic adsorption method, copper ions pre-coordinated with dopamine were added to Ti_3_C_2_ dispersion by rapid agitation, and then DSPE-PEG2000 was introduced to finally obtain Cu-loaded Ti_3_C_2_-Cu-PEG (Figure 1A). A molar ratio of Cu was measured about 0.3 wt% using inductively coupled plasma optical emission spectrometer (ICP-OES) measurements. Transmission electron microscopy (TEM) images (Figure 1B) show that Ti_3_C_2_-Cu-PEG nanocomposites have high Cu monodispersity with no obvious Cu nanoparticles detected, with an average diameter of about 200 nm. In addition, the energy dispersive X-ray spectroscopy (EDX)mapping (Figure 1C) shows that Cu (red), C (blue), and Ti (green) are uniformly distributed across the skeleton of the as-synthesized Ti_3_C_2_-Cu-PEG nanocomposites. High resolution TEM (HRTEM) image (Figure 1D) shows that Ti_3_C_2_-Cu-PEG nanocomposites have high crystallinity. The thickness of Ti_3_C_2_-Cu-PEG nanocomposites can be further characterized by atomic force microscopy (AFM) measurements. Through AFM image analysis (Figure 1E), the thickness of the obtained Ti_3_C_2_-Cu-PEG nanocomposites is relatively uniform, and the size is about 2 nm. The elemental composition and surface electronic states of Ti_3_C_2_-Cu-PEG nanocomposites are further characterized by X-ray photoelectron spectroscopy (XPS). Compared with Ti_3_C_2_-PEG, the measured spectra of Ti_3_C_2_-Cu-PEG nanocomposites show Cu (Figure 1F). The high-resolution XPS spectra of Cu2p at 933.1 eV and 952.8 eV correspond to Cu2p3/2 and Cu2p1/2, respectively (Figure 1G). In addition, the copper valence is composed of Cu^+^ (932.5, 952 eV)/Cu^2+^ (934.2, 955.2 eV), and the proportion of Cu^+^ is higher than that of Cu^2+^. Furthermore, X-ray powder diffraction (XRD) is used to analyze the crystal phases of Ti_3_C_2_-PEG and Ti_3_C_2_-Cu-PEG (Figure 1H). The results show that there is no significant difference between the crystal phases of Ti_3_C_2_-PEG and Ti_3_C_2_-Cu-PEG, indicating that copper does not form nanocrystals in Ti_3_C_2_ nanosheets. In summary, these results confirm that Cu is uniformly loaded on the surface of Ti_3_C_2_-PEG and has two valence states, Cu^+^ and Cu^2+^, which can catalyze the decomposition of hydrogen peroxide and consume GSH, contributing to the formation of excessive ROS and unbalance of the redox TME [44].

### 3.2 Photothermal property assessment

**Figure 2:**
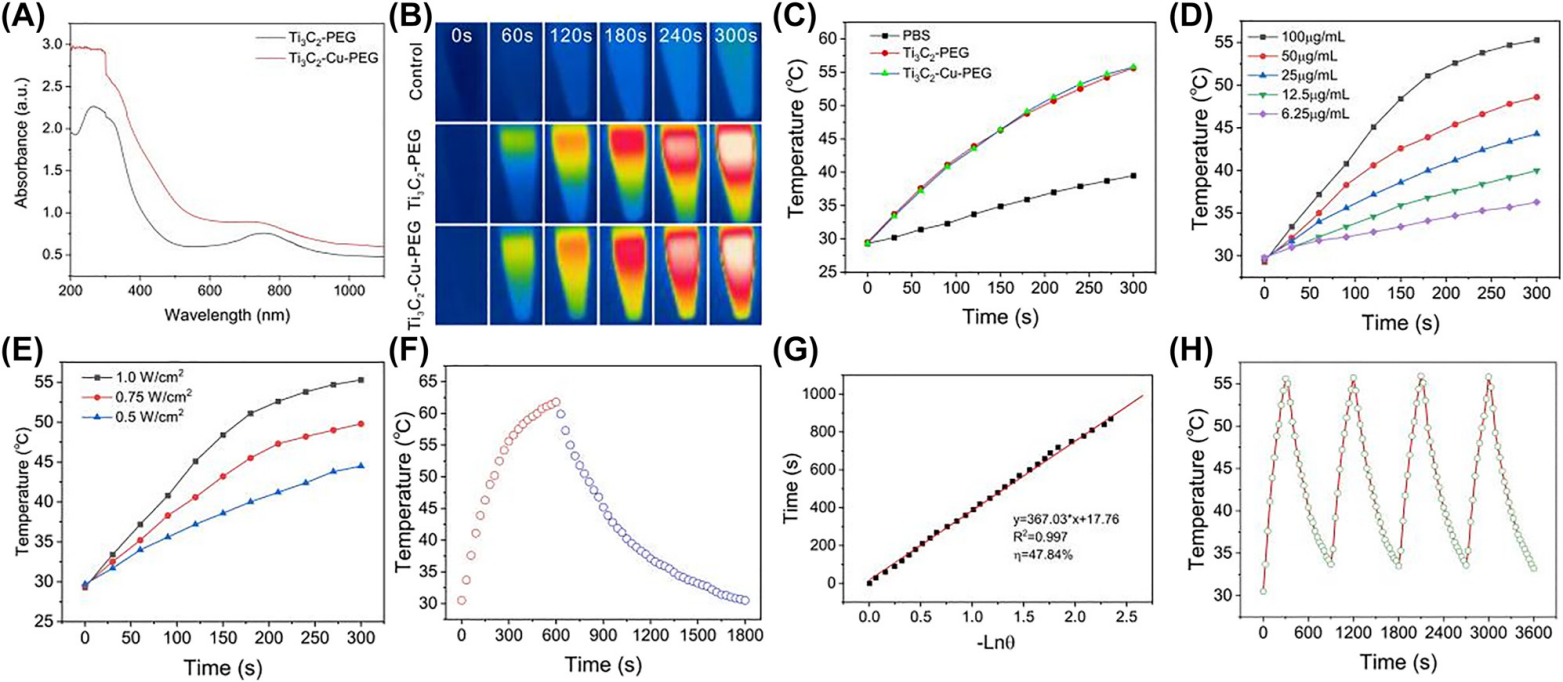
Photothermal performance of the Ti_3_C_2_-Cu-PEG. (A) UV-vis-NIR absorption spectra of Ti_3_C_2_-PEG and Ti_3_C_2_-Cu-PEG (100 μg/mL). (B) Infrared thermal images of the Ti_3_C_2_-Cu-PEG aqueous solution (100 μg/mL), Ti_3_C_2_-PEG (100 μg/mL) and PBS under irradiation by a 1064 nm laser with a power density of 1 W/cm^2^ for 5 min. (C) Temperature variation curves versus irradiation time for Ti_3_C_2_-Cu-PEG aqueous solution (100 μg/mL), Ti_3_C_2_-PEG aqueous solution (100 μg/mL) and PBS under laser irradiation (1064 nm, 1 W/cm^2^). (D) Temperature variation curves for Ti_3_C_2_-Cu-PEG aqueous solution with different concentrations under 1064 nm laser irradiation at 1 W/cm^2^. (E) Temperature variation curves of Ti_3_C_2_-Cu-PEG aqueous solution under varied irradiation powers (0.50, 0.75 and 1 W/cm^2^) at concentration of 100 μg/mL. (F) Photothermal effect of Ti_3_C_2_-Cu-PEG aqueous solution irradiated with a 1064 nm laser (1 W/cm^2^), in which the irradiation lasted for 600 s, and then the laser was shut off. (G) Linear time data versus -Lnθ obtained from the cooling period as shown in (F). (H) Recycling-heating profiles of the Ti_3_C_2_-Cu-PEG solution under 1064 nm laser irradiation at 1 W/cm^2^ for four laser on/off cycles.

The absorption spectra of Ti_3_C_2_-PEG and Ti_3_C_2_-Cu-PEG nanocomposites were studied by UV-vis-NIR absorption spectra. Compared with Ti_3_C_2_-PEG nanocomposites, the absorbance of Ti_3_C_2_-Cu-PEG nanocomposites is slightly increased in the NIR region and concentration-dependent (Figures 2A and S1), which is related to the doping of copper ions, indicating that Ti_3_C_2_-Cu-PEG nanocomposites was a desirable candidate as a photothermal agent for PTT at a wavelength of 1064 nm. The photothermal conversion properties of Ti_3_C_2_-Cu-PEG nanocomposites were investigated under 1064 nm irradiation with different concentrations. Temperature changes and corresponding images were recorded for Ti_3_C_2_-Cu-PEG nanocomposites irradiated with a 1064 nm laser (1 W/cm^2^) for 5 min (Figure 2B and C). A total temperature of 55.6 °C of the Ti_3_C_2_-Cu-PEG solution was observed after laser irradiation, while the temperature increase of PBS is negligible. Thus, the Ti_3_C_2_-Cu-PEG nanocomposite exhibited strong potential to act as effective photothermal agents (PTA) of NIR laser irradiation (1064 nm).

The temperature variations of Ti_3_C_2_-Cu-PEG nanocomposites solution with different concentrations upon 1064 nm laser irradiation at 1 W/cm^2^ for 5 min were investigated. As shown in Figure 2D, the temperature of Ti_3_C_2_-Cu-PEG nanocomposites increased up rapidly even at lower concentrations, and the higher the concentration the faster the warming rate. The power-dependent temperature elevation of Ti_3_C_2_-Cu-PEG nanocomposites (100 μg/mL) under 1064 nm laser irradiation was further recorded. As shown in Figure 2E, the temperature of Ti_3_C_2_-Cu-PEG nanocomposites solution dramatically increased with the laser power increasing from 0.5 W/cm^2^ to 1 W/cm^2^. Upon 1064 nm laser irradiation at 1 W/cm^2^ for 5 min, the temperature was achieved 55.8 °C, indicating the high photothermal capability of Ti_3_C_2_-Cu-PEG. In addition, photothermal conversion efficiency (*η*) is an essential parameter to evaluate a PTA. To calculate *η*, the temperature changes of Ti_3_C_2_-Cu-PEG nanocomposites (100 μg/mL, 1 mL) were recorded in a cycle of heating up and cooling (Figure 2E). By calculating the fitting data from the cooling stage, the *η* of Ti_3_C_2_-Cu-PEG was calculated to be approximately 47.84% using the method reported by Roper et al. [51], which was relatively higher than that of Au nanorods (21%) [52], Cu_9_S_5_ nanocrystals (25.7%) [53] and polypyrrole@Fe3O4 (39.15%) [54], as reported previously. In addition, after four cooling cycles, the nanosystem still had good photothermal properties, indicating its photothermal stability (Figure 2H). All these results indicated the great potential of Ti_3_C_2_-Cu-PEG nanocomposites for PTT.

### 3.3 *In vitro* ROS generation study

**Figure 3:**
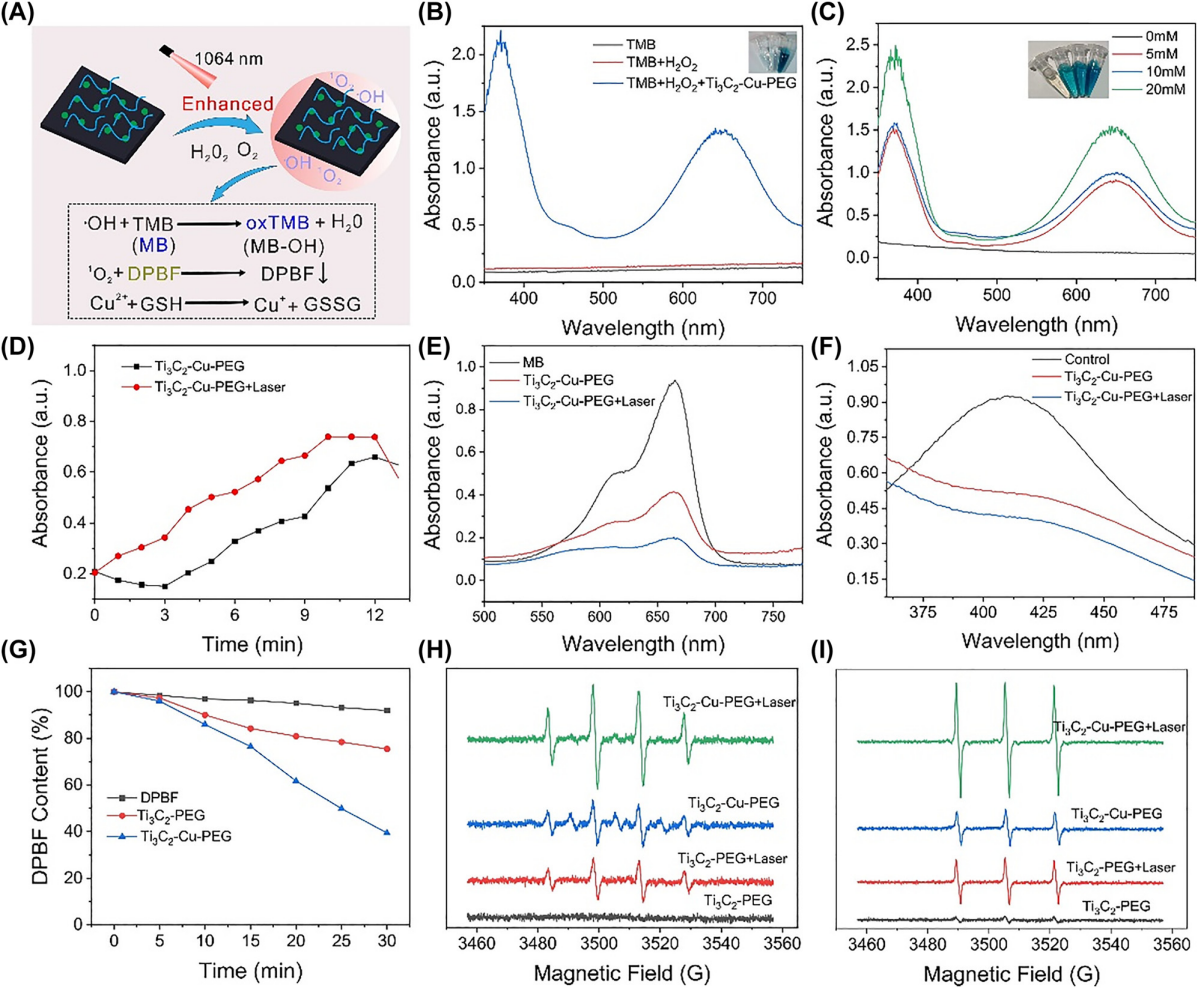
*In vitro *ROS generation. (A) Schematic presentation of ROS detection and GSH-depletion of Ti_3_C_2_-Cu-PEG. (B) UV–vis absorption spectra of the catalyzed oxidation of TMB (oxTMB) under different conditions. The insets are the corresponding visual color changes. (C) UV–vis absorption spectra of the oxidation of TMB (oxTMB) on Ti_3_C_2_-Cu-PEG with different H_2_O_2_ concentrations. The insets are the corresponding visual color changes. (D) Time-dependent absorbance changes at 652 nm of the oxidation of TMB for Ti_3_C_2_-Cu-PEG with or without laser irradiation. UV–vis absorption spectra of MB (E) and (F) GSH consumption after treated with Ti_3_C_2_-Cu-PEG with or without laser irradiation. (G) Depletion of DPBF due to ^1^O_2_ generation of Ti_3_C_2_-PEG and Ti_3_C_2_-Cu-PEG under laser irradiation. (H) ESR spectra of ·OH trapped by DMPO and ^1^O_2_ trapped by TEMP (I) with or without laser irradiation of Ti_3_C_2_-PEG and Ti_3_C_2_-Cu-PEG with or without laser irradiation (1064 nm, 1 W/cm^2^).

The successful fabrication of the Ti_3_C_2_-Cu-PEG nanocomposites encouraged us to investigate its multiple functions. Due to the presence of Cu, we speculated that the Ti_3_C_2_-Cu-PEG nanocomposites have the potential of effective catalytic activities to catalyze H_2_O_2_ or O_2_ under laser irradiation, and produce highly toxic ROS (·OH or ^1^O_2_) (Figure 3A) [55]. Cu^2+^ depletes GSH in the tumor microenvironment and further promotes ROS effects (Figure 3A). As shown in Figure 3B, the absorbance of TMB alone and TMB + H_2_O_2_ can be neglected, indicating that no oxidation reaction has occurred. After adding Ti_3_C_2_-Cu-PEG nanocomposites, the color of the mixture changed obviously. The two characteristic absorption peaks at 370 and 652 nm are generated by oxTMB, indicating that Ti_3_C_2_-Cu-PEG nanocomposites can induce the decomposition of H_2_O_2_ to form ·OH. Then, different concentrations of H_2_O_2_ (0, 5, 10, and 20 × 10^−3^ M) were used as substrates in acidic TMB solution (pH6.5) in weakly acidic phosphate buffered saline (PBS) (Figure 3C). The results showed that the formation of ·OH increased gradually with the increase of hydrogen peroxide concentration, which was also confirmed by embedded digital photographs. There were significant differences in absorbance between different treatment groups. As shown in Figure 3D, the ability of Ti_3_C_2_-Cu-PEG nanocomposites to generate ·OH under laser irradiation is stronger than that without laser irradiation, indicating that the photothermal effect enhances the catalytic reaction efficiency of Ti_3_C_2_-Cu-PEG nanocomposites. Further, ·OH production capacity was determined by degrading methylene blue (MB). As indicated in Figures 3E and S2, after Ti_3_C_2_-Cu-PEG treatment, the decrease of MB content in the treatment group with laser irradiation was higher than that in the non-laser treatment group. These results indicate that Ti_3_C_2_-Cu-PEG nanocomposites can effectively induce the decomposition of H_2_O_2_ to ·OH, and laser irradiation can promote this reaction.

In the tumor microenvironment, GSH is an overexpression endogenous antioxidant that maintains tumor cellular redox homeostasis and inhibits ROS-induced cell damage. Based on the hyperthermia enhanced catalytic reactions, the ability to induce GSH depletion was also examined (Figure 3F). Under laser irradiation, GSH was consumed by Ti_3_C_2_-Cu-PEG nanocomposites. As expected, high temperatures induced by laser irradiation may result in greater GSH depletion under mildly acidic conditions. At the same time, 1, 3-diphenylisobenzofuran (DPBF) indicator was used to detect the formation of ^1^O_2_. The typical characteristic peak of DPBF gradually decreases at 410 nm because ^1^O_2_ reacts with DPPF to form o-dibenzoylbenzene. As shown in the Figures 3G and S3, at 1064 nm laser irradiation, the characteristic peak of DPBF decreased faster than that of the non-irradiated group, and Ti_3_C_2_-Cu-PEG nanocomposites could play a photothermal and photodynamic role. In addition, electron spin resonance (ESR) was also measured to further demonstrate the formation of ·OH and ^1^O_2_ (Figure 3H and I). The production of ·OH and ^1^O_2_ from Ti_3_C_2_-Cu-PEG nanocomposites was significantly enhanced under laser irradiation than that without laser irradiation. Therefore, these results suggest that GSH depletion and photothermal accelerated ROS production, thereby disrupting intracellular redo homeostasis and enhancing ROS killing effect on tumor cells.

### 3.4 Cellular experiments

**Figure 4:**
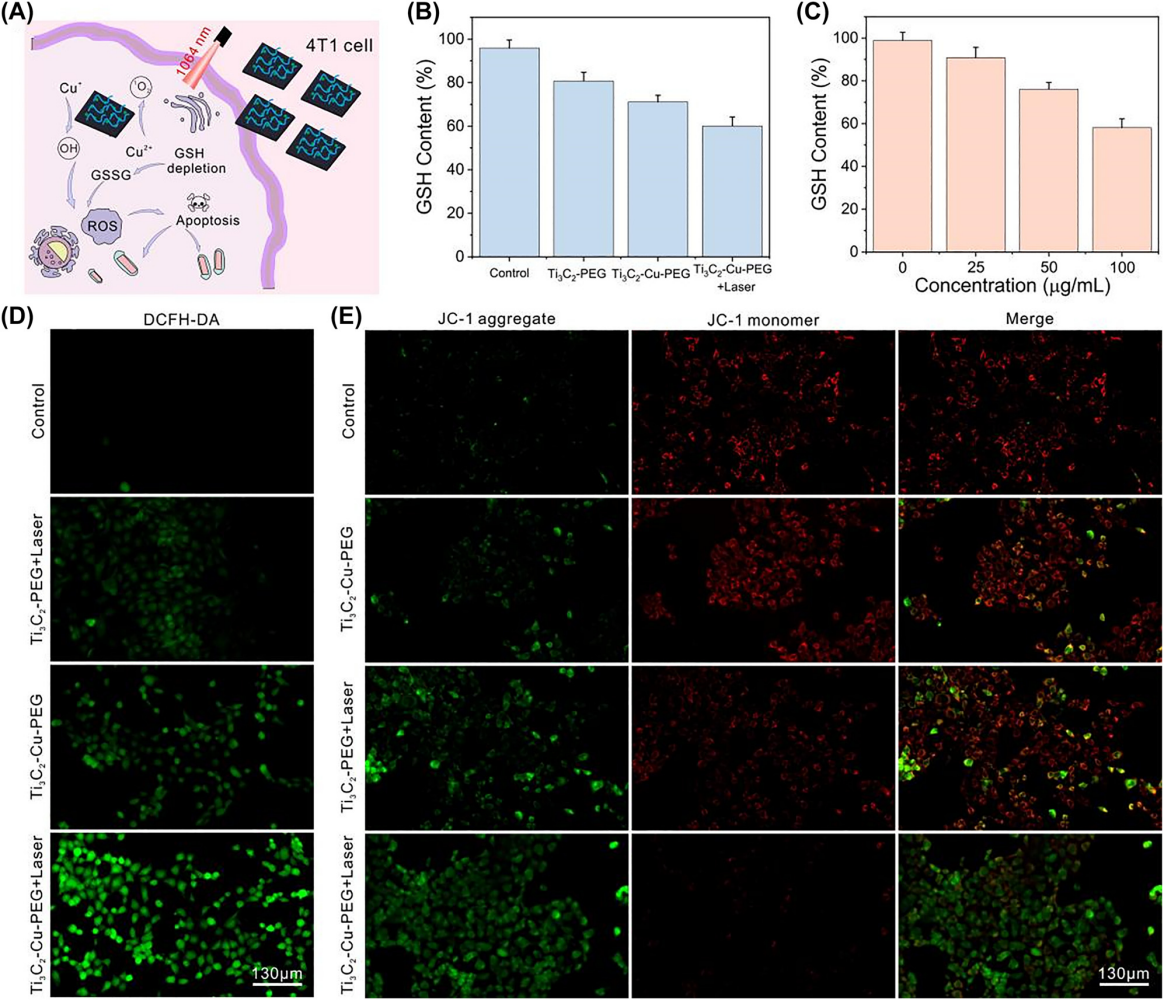
Intracellular ROS generation and GSH depletion. (A) Schematic illustration of ROS-generation by Ti3C2-Cu-PEG-PEG in 4T1 cells. (B) Intracellular GSH levels after various treatment. (C) Intracellular GSH levels after treatment with Ti_3_C_2_-Cu-PEG at different concentrations. Fluorescence images of 4T1 cells stained with DCFH-DA (D), and JC-1 (E) after various treatments, respectively. (1064 nm, 1.0 W/cm^2^, 5 min; Scale bar: 130 μm).

It was further verified that the generation of ROS photothermally enhanced led to the apoptosis of tumor cells. The reaction of Ti_3_C_2_-Cu-PEG nanocomposites in 4T1 cells under irradiation was shown in Figure 4A. To confirm GSH depletion, GSH and GSSG assay kits were used to measure intracellular GSH concentrations after Ti_3_C_2_-Cu-PEG nanocomposites treatment. As shown in Figure 4B, compared with the control group and Ti_3_C_2_-PEG nanocomposites group, the intracellular GSH decreased significantly after Ti_3_C_2_-Cu-PEG nanocomposites treatment, and the GSH decreased more in the Ti_3_C_2_-Cu-PEG nanocomposites group after laser irradiation. Under laser irradiation, the GSH level of 4T1 cells treated with different concentrations of Ti_3_C_2_-Cu-PEG nanocomposites gradually decreased, indicating a dose-dependent pattern (Figure 4C), which was related to photothermal and copper content of Ti_3_C_2_-Cu-PEG.

As shown in Figure 4D, 2′,7′-dichlorodihydrofluorescein diacetate (DCFH-DA) staining assay was used evaluate intracellular ROS generation. DCFH-DA can react with ROS to produce 2′,7′-dichlorodihydrofluorescein (DCF) with green fluorescence. The weak green fluorescence was shown in the control group and Ti_3_C_2_-PEG + Laser treatment group. Furthermore, 4T1 cells treated with Ti_3_C_2_-Cu-PEG nanocomposites with 1064 nm laser irradiation indicated a stronger fluorescence compared with the groups without laser irradiation, indicating that Ti_3_C_2_-Cu-PEG nanocomposites could produce a large amount of ROS and the photothermal effect enhanced the production of ROS. Therefore, Ti_3_C_2_-Cu-PEG nanocomposites can initiate effective NDT against tumor cells under laser irradiation. Further, the effect of ROS on cell apoptosis was explored. Because highly correlated with mitochondrial dysfunction, JC-1 method was used to study mitochondrial membrane potential in different treatment groups [56]. As shown in Figure 4E, compared with the control, Ti_3_C_2_-Cu-PEG without laser and Ti_3_C_2_-PEG with laser irradiation, Ti_3_C_2_-Cu-PEG laser irradiation group had the largest change in membrane potential, which was consistent with the above results, indicating that Ti_3_C_2_-Cu-PEG nanocomposites produced a large amount of ROS to cause cell apoptosis.

**Figure 5:**
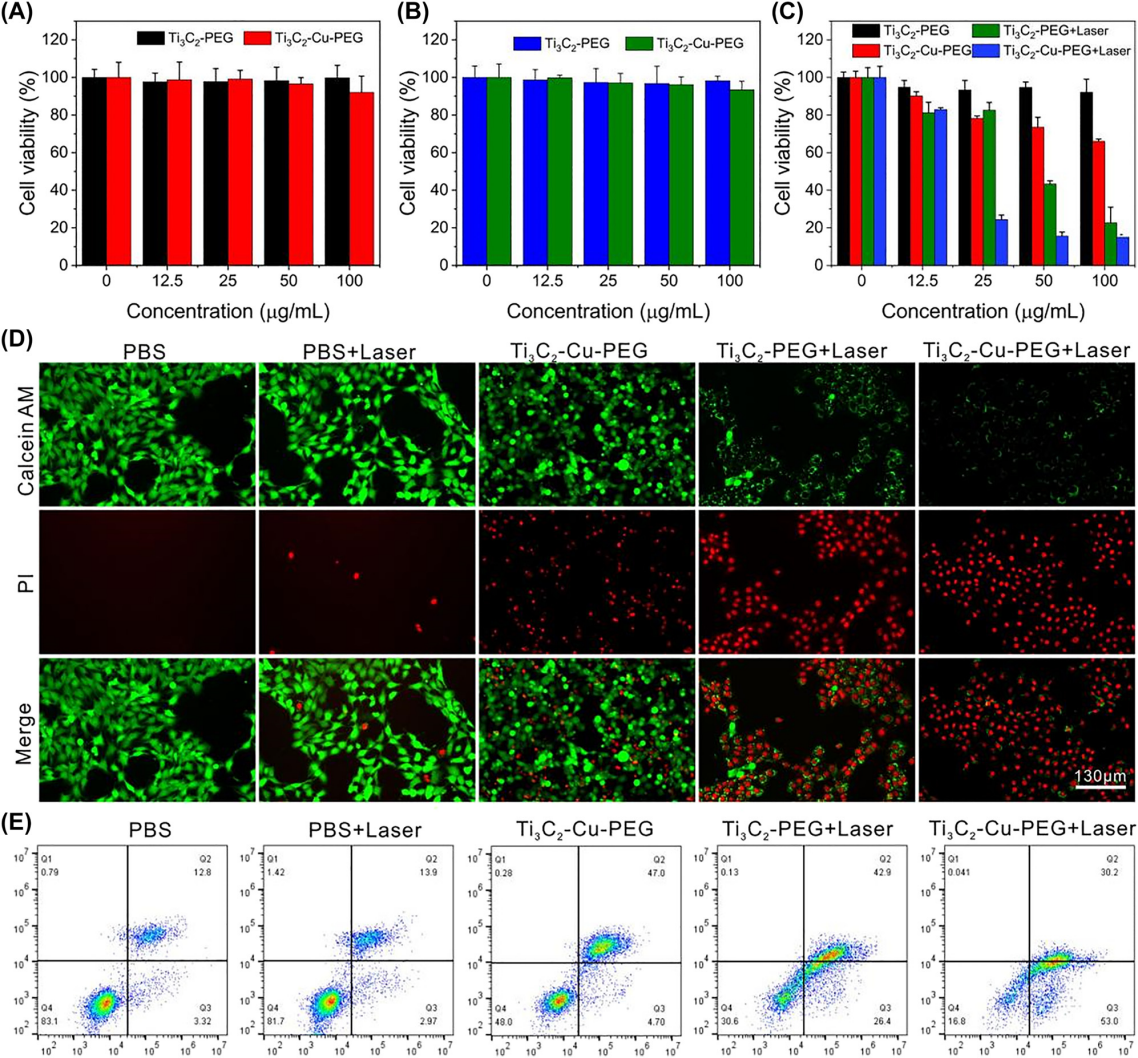
*In vitro* evaluation of the cytotoxicity for cancer cells under NIR irradiation. Relative cell viabilities of HUVEC cells (A), and NIH3T3 cells (B) incubated with Ti_3_C_2_-PEG and Ti_3_C_2_-Cu-PEG at diverse concentrations (0, 12.5, 25, 50, 100 μg/mL). (C) Cytotoxicity profiles of 4T1 cells after being treated with different formulations. (D) Calcein-AM/PI staining and, (E) flow cytometry analysis of cell apoptosis of 4T1 cells incubated with different formulations.

Subsequently, encouraged by the result of ROS-mediated cell apoptosis *in vitro*, the cytotoxicity of the Ti_3_C_2_-Cu-PEG nanocomposites was further evaluated. Normal cells (HUVEC and NIH3T3) and cancer cells (4T1) incubated with different concentrations of Ti_3_C_2_-Cu-PEG were investigated by CCK-8 assay. The survival rates of HUVEC and NIH3T3 cells treated with Ti_3_C_2_-PEG and Ti_3_C_2_-Cu-PEG were above 90%, indicating that the toxicity of the material to normal cells was negligible. These results confirm that Ti_3_C_2_-Cu-PEG nanocomposites have high biocompatibility and low toxicity, and could be used for therapeutic applications *in vivo*. The *in vitro* therapeutic effect was further evaluated in 4T1 cells. Ti_3_C_2_-Cu-PEG nanocomposites can produce ROS in the tumor microenvironment under irradiation and is specificity for killing cancer cells. For the cytotoxicity assay of 4T1 cells, the cytotoxicity increased with the increase of concentration. As shown in Figure 5C, compared with Ti_3_C_2_-PEG under 1064 nm irradiation and only Ti_3_C_2_-Cu-PEG treatment groups, more 4T1 cells have been killed after incubation with Ti_3_C_2_-Cu-PEG under 1064 nm irradiation. The apoptosis of 4T1 cells is caused by a large amount of intracellular ROS production and photothermal therapy.

In addition, double staining experiments with calcein AM (green) and propidium iodide (red) further confirmed the effective killing effect of Ti_3_C_2_-Cu-PEG nanocomposites on 4T1 cells by laser irradiation (Figure 5D). Flow cytometry apoptosis assay showed that photothermal and chemokinetic treatment after Ti_3_C_2_-Cu-PEG staining resulted in more cell death with Annexin V-FITC and propidium iodide (PI). The apoptotic ratio induced by Ti_3_C_2_-Cu-PEG under 1064 nm irradiation, 83.2% (Q2 + Q3), is markedly higher than that of Ti_3_C_2_-Cu-PEG (51.7%) and Ti_3_C_2_-PEG under 1064 nm irradiation (69.3%) under the same conditions, which are composed of early apoptosis and late apoptosis (Figure 5E). These *in vitro* results clearly manifest that the Ti_3_C_2_-Cu-PEG nanocomposites could achieve efficient synergistic killing to cancer cells.

### 3.5 *In vivo* antitumor research

**Figure 6:**
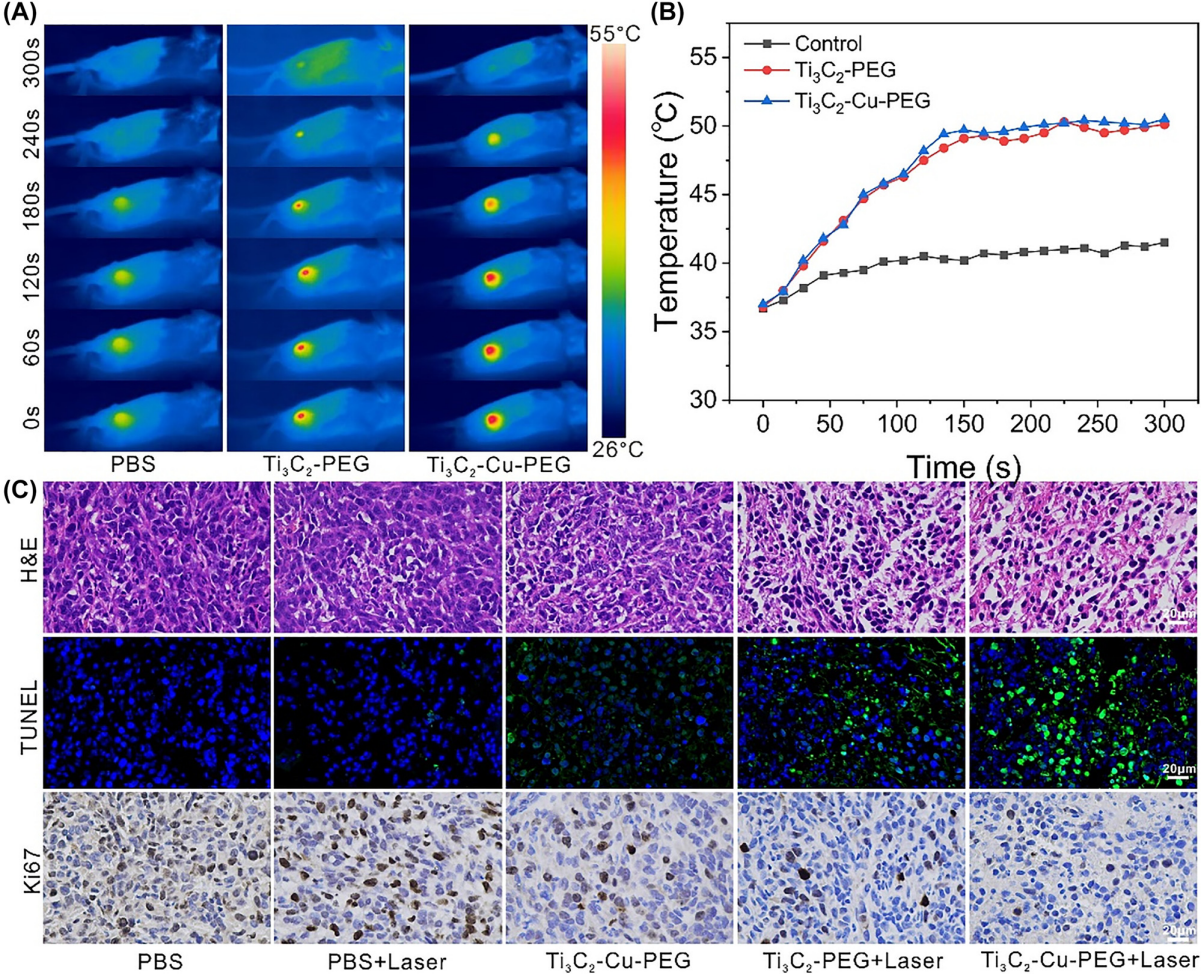
*In vivo *infrared thermography and tumor slices. (A) Infrared thermal images of 4T1 tumor-bearing mice and (B) temperature profiles of the tumors after intratumor injection of Ti_3_C_2_-PEG and Ti_3_C_2_-Cu-PEG under laser irradiation (1064 nm, 1 W/cm^2^) for 5 min. (C) H&E, TUNEL and Ki67 staining images of tumor slices collected from different groups 24 h post treatments.

*In vitro* cell experiments showed that Ti_3_C_2_-Cu-PEG nanocomposites could effectively kill 4T1 cells by synergistic GSH-depletion and photothermal-enhanced nanodynamic therapy, indicating that Ti_3_C_2_-Cu-PEG nanocomposites has further application prospects *in vivo*. Subsequently, to evaluate the effect of Ti_3_C_2_-Cu-PEG treatment, PBS, Ti_3_C_2_-PEG and Ti_3_C_2_-Cu-PEG were injected into the tumors of 4T1 tumor-bearing mice *in situ*. Then, the tumor was irradiated with a laser (1064 nm, 1 W/cm^2^) for 5 min at 4 h post administration to ensure nanocomposites taken up by the tumor cell. Real-time and spatial temperature profiles at tumor sites in mice were recorded with an infrared thermal imaging camera (Figure 6A and B). The results showed that the tumor temperature of Ti_3_C_2_-PEG nanocomposites and Ti_3_C_2_-Cu-PEG nanocomposites increased rapidly to 50 °C within 300 s, while the tumor temperature of PBS group increased to about 40 °C, indicating that Ti_3_C_2_-PEG and Ti_3_C_2_-Cu-PEG had good photothermal effects. In contrast, a slight increase in tumor temperature in the PBS-treated group did not have a killing effect on tumor cells.

To further explore the detailed treatment mechanisms of the material, tumor tissues were collected 24 h after laser treatment for hematoxylin and eosin staining (H&E), terminal deoxynucleoside transferase dUTP incision end labeling (TUNEL) and Ki67 antibody staining (Figure 6C).

The results of H&E staining showed that the laser-irradiated Ti_3_C_2_-PEG and Ti_3_C_2_-Cu-PEG groups had severe tumor cell damage, and a large number of TUNEL-positive cells were found in the TUNEL staining images, which showed destructive necrosis and apoptosis of tumor cells. In contrast, no or only minor tumor damage was observed in the other treatment groups. Ki67 antibody staining was shown that the proliferative activity of tumor cells was inhibited of Ti_3_C_2_-Cu-PEG treatment with 1064 nm laser, while the PBS group has hardly significant adverse effect on cell proliferation. These results together demonstrated the efficient synergistic effect of Ti_3_C_2_-Cu-PEG in tumor.

**Figure 7:**
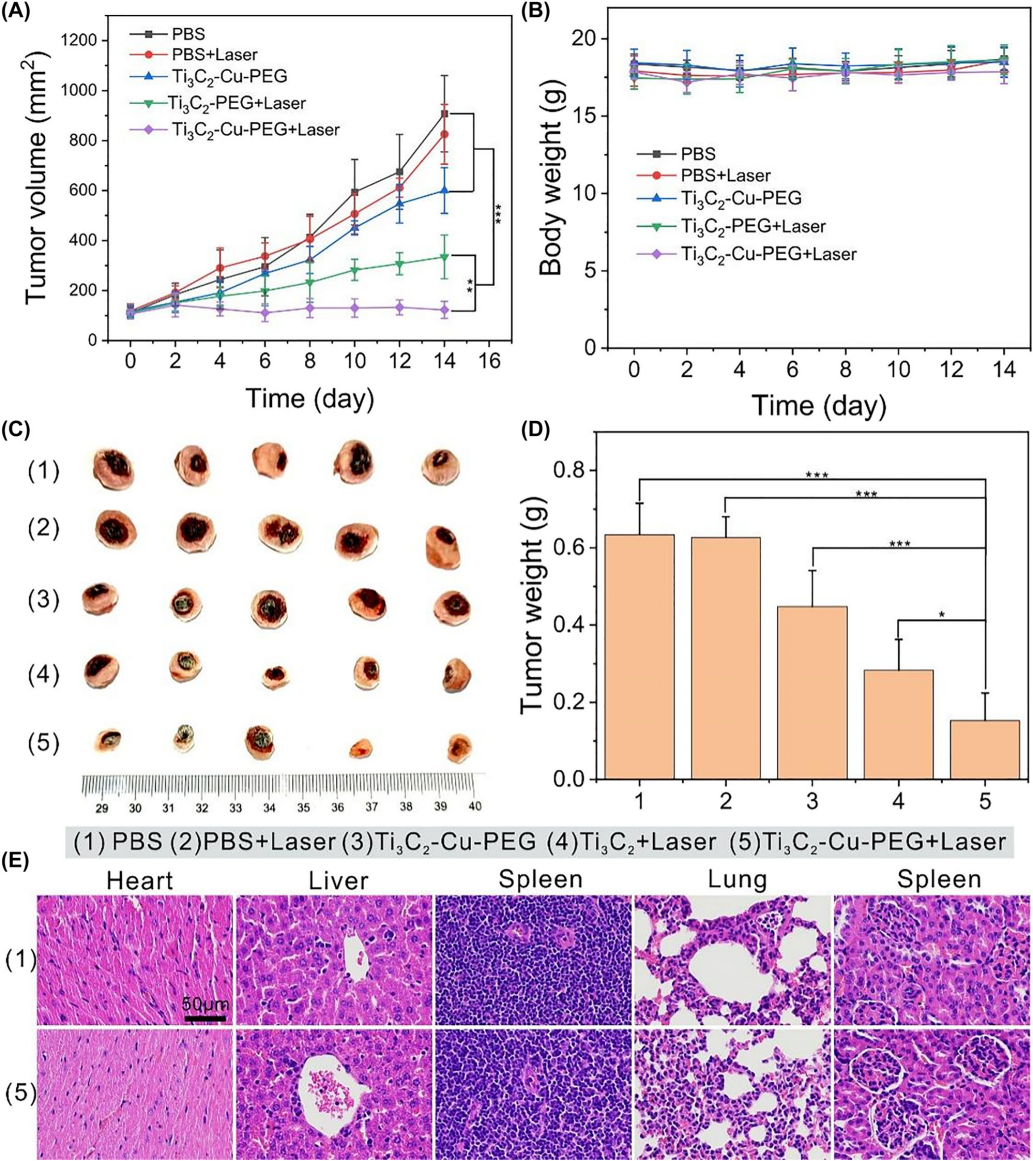
*In vivo *synergistic GSH-depletion and photothermal-enhanced nanodynamic therapy. (A) The tumor volume curves, and (B) body weight curves of mice in various groups (*n* = 5). (C) Digital photos of isolated tumors from different groups after treatments on 14th. (D) Tumor weights after treatments. (E) H&E staining of major organs from PBS and TTi_3_C_2_-Cu-PEG 14 days after treatment. (Scale bar: 50 μm).

Inspired by the above results, we investigated the antitumor effect of Ti_3_C_2_-Cu-PEG nanocomposites *in vivo* with more details. To this end, 4T1 tumour-bearing mice were randomly divided into 5 groups (*n* = 5 for each group): PBS, PBS + Laser, Ti_3_C_2_-Cu-PEG, Ti_3_C_2_-PEG + Laser and Ti_3_C_2_-Cu-PEG + Laser. 4 h after intratumoral administration, the tumor was irradiated with laser for 5 min (1064 nm, 1 W/cm^2^). Tumor size and animal weight were measured every other day (Figure 7A and B). Compared with PBS treatment group, Ti_3_C_2_-PEG + Laser and Ti_3_C_2_-Cu-PEG + Laser showed significant tumor suppressive effect, the latter was stronger than the former (*p* < 0.05), indicating that the synergistic effect of Ti_3_C_2_-Cu-PEG was higher (Figure 7A). In addition, the tumor inhibition in the Ti_3_C_2_-Cu-PEG + Laser group was significantly stronger than that in the Ti_3_C_2_-Cu-PEG group (Figure 7A), indicating that the synergistic GSH-depletion and photothermal-enhanced nanodynamic therapy was more effective than either therapy catalyzed alone.

In addition, weight fluctuations in treated mice were negligible. After 14 days, the mice were sacrificed, and the tumors were photographed and weighed. The results showed that Ti_3_C_2_-Cu-PEG + Laser group had the best treatment effect (Figure 7C and D), which was consistent with the above results (Figure 7A). Some blood biochemical parameters were tested to investigate, the aspartate aminotransferase (AST), alkaline phosphatase (ALP), alanine aminotransferase (ALT), urea nitrogen (BUN), and creatinine (CREA) were within the normal range, indicating the negligible adverse effects on the liver and kidneys (Figure S4). Major organs were stained for H&E to investigate the biosafety of Ti_3_C_2_-Cu-PEG *in vivo* (Figures 7E and S5). These analyses showed that neither Ti_3_C_2_ nor Ti_3_C_2_-Cu-PEG exhibited significant toxicity. These results further demonstrate the relative superiority of Ti_3_C_2_-Cu-PEG, which can effectively inhibit tumor growth without detecting systemic toxicity.

## 4 Conclusions

In conclusion, we have successfully fabricated a Ti_3_C_2_-Cu-PEG nanosystem for NIR-II light-induced tumor synergistic therapy via a facile and rational strategy. With ultrasmall copper species uniformly loaded on the two-dimensional Ti_3_C_2_ nanosheets, this nanosystem not only exhibited the optical and chemical properties of MXene and Cu cations, but also proved much higher stability than the counterpart MXene and Cu species, laying good foundation for elongated treatment. Upon the irradiation of NIR-II light, Ti_3_C_2_-Cu-PEG would initiate the generation of a large amount of highly toxic ROS and depletion of GSH, heat the neighboring environment to promote these two processes, achieving highly effective antitumor therapy. Both *in vitro* and *in vivo* studies have confirmed that Ti_3_C_2_-Cu-PEG can significantly inhibit the growth of the tumor, and this material showed good biocompatibility and negligible toxicity *in vivo*. This work suggests that smart design of NIR-II light-responsive nanomaterials with TME-stimulated simultaneous NDT and light-responsive nanomaterials GSH depletion provides a new approach for highly efficient cancer treatment.

## Supplementary Material

Supplementary Material Details
