# Supplementary material for: Copper decorated Ti3C2 nanosystem with NIR-II-induced GSH-depletion and reactive oxygen species generation for efficient nanodynamic therapy
Source: Nanophotonics. 2022 Nov 4;11(22):5189–204. doi: 10.1515/nanoph-2022-0599 (PMC11501824; doi:10.1515/nanoph-2022-0599)
Supplement: Supplementary file 1 — Supplementary Material Details [file j_nanoph-2022-0599_suppl.docx]

**Supporting Information**

**Copper [Decorate](javascript:;)d Ti_3_C_2_ Nanosystem with NIR-II-induced GSH-Depletion and Reactive Oxygen Species Generation for Efficient** **Nanodynamic Therapy**

*Yuanyuan Zhang,^1^ Shuang Li,^1^ Xueyang Fang,^1^ Beiping Miao,^1^ Yujie Wang,^1,2^ Jiantao Liu*,^3^ Guohui Nie,*^1,2^ Bin Zhang*^1,2^*

^1^ Shenzhen Key Laboratory of nanozymes and Translational Cancer Research, Department of Otolaryngology, Shenzhen Institute of Translational Medicine, The First Affiliated Hospital of Shenzhen University, Shenzhen Second People's Hospital, Shenzhen 518035, China.

^2^ State Key Laboratory of Chemical Oncogenomics, Guangdong Provincial Key Laboratory of Chemical Genomics, Peking University Shenzhen Graduate School, Shenzhen, 518055, China.

^3^ Department of Orthopedics, The First Hospital of Xi'an Jiaotong University, Xi'an, 710061.

Corresponding Authors:

Dr. Jiantao Liu: [liujiantao2010xjtu@163.com](mailto:liujiantao2010xjtu@163.com)

Prof. Guohui Nie: [nieguohui@email.szu.edu.cn](mailto:nieguohui@email.szu.edu.cn)

Prof. Bin Zhang: [wenwubin09@163.com](mailto:wenwubin09@163.com)


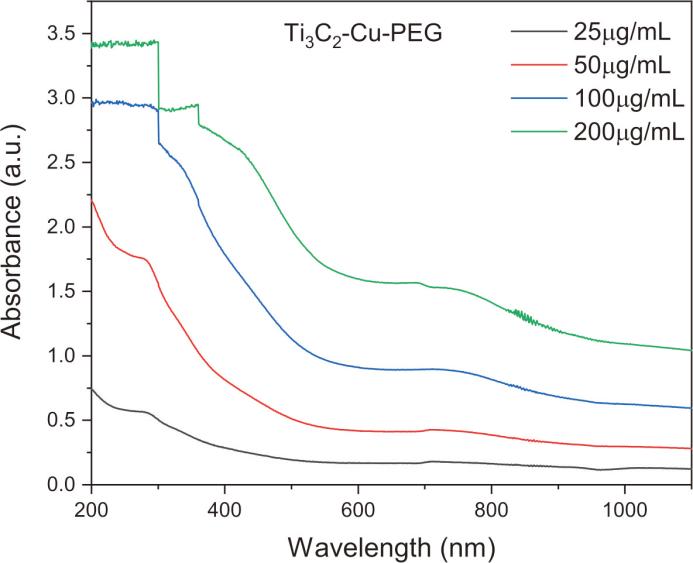


Figure S1. UV-vis-NIR absorption spectra of Ti_3_C_2_-Cu-PEG at different concentrations.


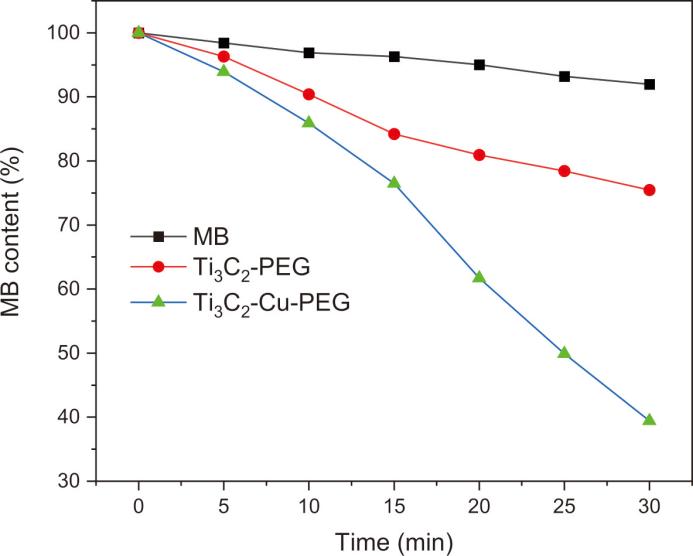


Figure S2. Depletion of MB due to ∙OH generation of Ti_3_C_2_-Cu-PEG under laser irradiation (1064 nm, 1 W/cm^2^).


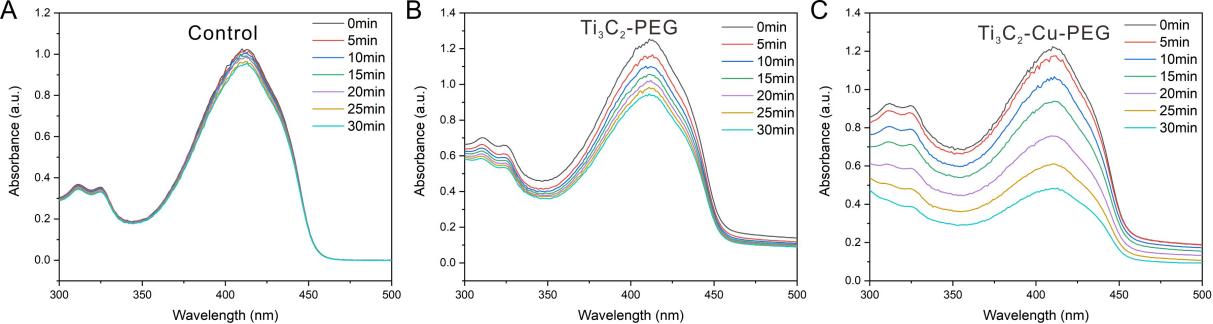


Figure S3. Absorption spectrum changes of DPBF with Control (A), Ti_3_C_2_-PEG (B) and Ti_3_C_2_-Cu-PEG (C) under laser irradiation (1064 nm, 1 W/cm^2^).


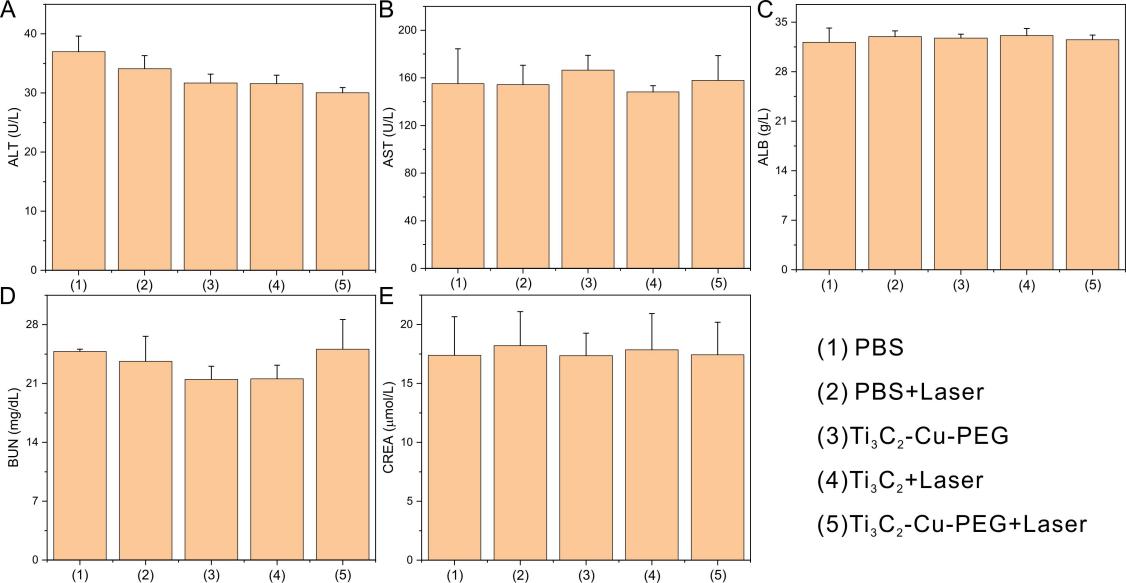


Figure S4. Blood biochemical analysis of BALB/c mice after the intravenous administration with different composites at 14^th^ day.


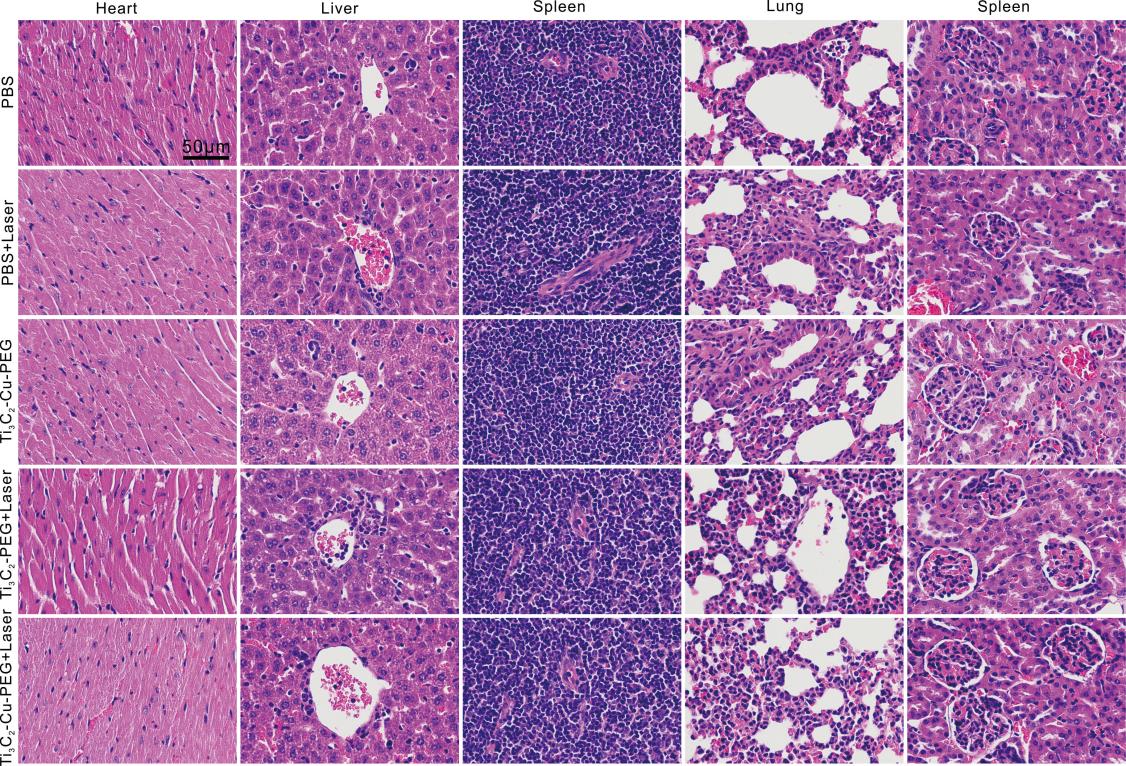


Figure S5. H&E-stained tissue sections of major organs from BALB/c mice in biosafety experiments (Scale bar: 50 µm).
